# Functional and MRI follow-up after reconstruction of chronic ruptures of the Achilles tendon Myerson type III using the triple-loop plantaris tendon wrapped with central turndown flap: a case series

**DOI:** 10.1186/s13018-015-0256-y

**Published:** 2015-07-15

**Authors:** Ahmed F. Sadek, Ezzat H. Fouly, Mohammed A. Laklok, Mohammed F. Amin

**Affiliations:** Orthopaedic Surgery Department, Minia University Hospital, Minia, Egypt; Radiology Department, Minia University Hospital, Minia, Egypt

**Keywords:** Chronic Achilles tendon ruptures, Triple-loop plantaris, Turndown flap, MRI

## Abstract

**Background:**

Reconstruction of chronic ruptures of the Achilles tendon poses a great challenge for the orthopaedic surgeon both technically and functionally. The aim of this study was to assess the results of a new technique for reconstruction of chronic Achilles tendon ruptures with defects longer than 5 cm using the triple-loop plantaris tendon autograft wrapped in a central turndown flap from the proximal portion of the Achilles tendon.

**Patients and methods:**

Eighteen patients (14 female and 4 male; mean age, 40.7 years), having chronic ruptures of the Achilles tendon Myerson type III, were enrolled in this study. The mean follow-up period of our patients was 21.8 months. All patients were assessed via the following parameters: lag of interference since the first complaint, length of the defect, length of the turndown flap and length of the harvested plantaris tendon, surgery time, complications, active range of motion at the ankle and the final score. Average values were presented as means. Independent sample *t* test, Mann Whitney test, paired sample *t* test and Pearson’s correlation coefficient were used to evaluate the clinical and functional results. The results were considered statistically significant if a *P* value was <0.05. To analyse the time course of the gap following surgery, the data from the first MRI session were compared with those from the second and third sessions using the Wilcoxon’s signed rank test. In addition, the paired data of the tendon gap disappearance rate between T1-weighted and T2-weighted images were also compared using the McNemar test.

**Results:**

The mean preoperative American Orthopaedic Foot and Ankle Society score was 62.2 points while at the patients’ last follow-up, the mean postoperative score was 94.9 points. The results of this study confirmed that both the Achilles tendon healing and tendon gap disappearance have been perceived with higher sensitivity in T2-weighted images than in T1-weighted images.

**Conclusions:**

We believe that this new technique is biologic, synchronous and reliable in cases of chronic Achilles tendon ruptures with defects longer than 5 cm.

## Background

Chronic ruptures of the Achilles tendon are those ruptures presented to treatment after 4–6 weeks of injury. Those injuries result from strenuous sports activities, systemic disorders, or insertional tendinopathies [1]. Chronic ruptures eventually result in retraction of the proximal portion of the tendon with subsequent biomechanical disadvantage of the triceps suri. This subsequently results in both weak active plantar flexion of the ankle and non-propulsive gait on the affected side [1].

Diagnosis of a chronic rupture of the Achilles tendon requires a high index of suspicion. That is verified by the fact that 20–25 % of cases of the Achilles tendon rupture are initially missed or misdiagnosed [2, 3]. This is owed to the significant decrease in the magnitude of pain after the initial trauma in addition to the active plantar flexion of the ankle recruited by either accessory plantar flexors, fibrous healing of the tendon or plantaris tendon [3]. Clinically, the patient complains of acute pain at the heel, inability to climb stairs or stand tiptoe.

In Myerson’s classification [4], Achilles tendon ruptures were classified according to the magnitude of the defect into three categories: (I) the defect measures 1 and 2 cm, (II) the defect measures 2–5 cm, and (III) the defect measures >5 cm. Most surgeons encourage operative treatment for chronic ruptures when possible to restore proper active plantar flexion of the ankle [1]. In Myerson type III, the surgeon is confronted with degenerated and frayed tendinous tissue which, after debridement, magnifies the defect. Ideally, the technique proposed for reconstruction of a chronic Achilles tendon rupture should be both simple and less morbid. The aim of such procedure should be retaining normal functional capacity within a relatively short time [5, 6].

In this study, the authors present a new technique for reconstruction of chronic ruptures of the Achilles tendon Myerson type III using the triple-loop plantaris tendon autograft wrapped in a central turndown flap from the proximal portion of the Achilles tendon. The aim of this technique is to provide powerful, biological, synchronous and less adherent reconstruction. This study aims at evaluation of both the functional and radiological outcomes with special emphasis on MRI.

## Patients and methods

Eighteen patients (14 female and 4 male), having chronic ruptures of the Achilles tendon Myerson type III, were prospectively studied. The mean age of patients enrolled in this study was 40.7 years (range, 21–64). Fourteen right and four left Achilles tendons were affected. Human ethical committee approval from Minia University Hospital was obtained in December 2008. A full informed consent was obtained from all patients involved in the study. All patients have been operated in our centre in the period from January 2009 to June 2013.

*Inclusion criteria* included the following: a rupture of the Achilles tendon with a resultant defect longer than 5 cm for more than 6 weeks in skeletally mature patients who were proven radiologically to have plantaris tendon. *Exclusion criteria* were as follows: patients who were skeletally immature, medically unfit, having any local vascular or skin problem at the foot or ankle and those who had no plantaris tendon.

### Preoperative evaluation

#### Clinical evaluation

A thorough history taking and clinical evaluation of all patients were performed with special emphasis on the following: the presence of tendon gap and the positivity of (Thompson) calf squeeze test. All patients were evaluated regarding the active range of motion (ROM) of the ankle and the degree of calf atrophy in comparison to the healthy side. In addition, functional evaluation using American Orthopaedic Foot and Ankle Society (AOFAS) ankle and hind foot scoring system [7] was performed both preoperatively and postoperatively at the 3rd, 6th and 12th months.

#### Radiological evaluation

All patients were subjected to preoperative routine ankle radiographs in anteroposterior and lateral projections to confirm the presence of Haglund’s deformity, or Achilles tendon calcifications.

##### MRI protocol

All patients were subjected to MRI examination 1 week preoperatively and then postoperatively at the end of 4th, 8th and 12th weeks. The aim of preoperative MRI was defining the diagnosis, assessing the size of the defect, and confirming the presence of plantaris tendon. The authors used a 1.5-T MRI system (Gyro scan Intera, Philips medical Systems, Netherlands) with a commercially available quadrature cervical spinal coil or knee coil. During MRI, the patient lay in the supine position with the affected Achilles tendon placed on the coil. The protocols were sagittal and axial fat-suppressed spin-echo T1-weighted images (TR/TE, 660/17), axial, and sagittal fast spin-echo T2-weighted images (3500/84). Axial images were obtained with a 4-mm section thickness, 2-mm gap, 15 × 15 cm field of view, 256 × 192 matrix, and two signals averaged. Sagittal images were obtained with a 3-mm section thickness, 1-mm gap, 15 × 15 cm field of view, 256 × 192 matrix, and two signals averaged. No contrast was administered.

##### MRI evaluation

Images were evaluated by the same musculoskeletal radiologist who was blinded to the clinical data. The assessments were compared with the surgical and clinical findings.

##### MRI interpretation for the presence or absence of tendon gap and gap length

**Fig. 1:**
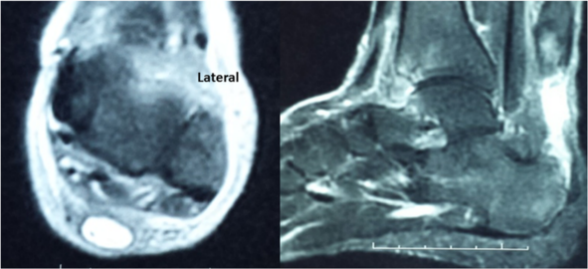
Preoperative MR images axial T2 fat sat. (*left*) and sagittal T2 fat sat. (*right*) show a complete tear of the Achilles tendon with a gap about 2.4 cm filled with fluid signal intensity

Only axial images were used for tendon gap evaluation as the sagittal images could not be used because of the brush-like appearance of the ruptured tendon ends (Fig. 1). A tendon gap was confirmed in three situations. First, when no area of hypointensity comparable with that of the neighbouring tendon was visible in the space that had probably been occupied by the Achilles tendon before rupture. Second, if the hypointense area within the contour was macroscopically smaller than 1/4 of the contour. Third, if the areas of hypointensity had a dotted distribution. Gap length was calculated by multiplying 6 mm (the sum of the slice thickness and the slice gap) by the number of axial images showing a tendon gap. Gap lengths thus were in multiples of 6 ± 2 mm, considering error in the first and last slices. If an area showing the contour of the Achilles tendon was visible on all axial images at the rupture site, this area was referred to as the compartment of the Achilles tendon, whether or not the signal intensity within this area was low enough to indicate the presence of Achilles tendon fibres.

### Surgical technique

**Fig. 2:**
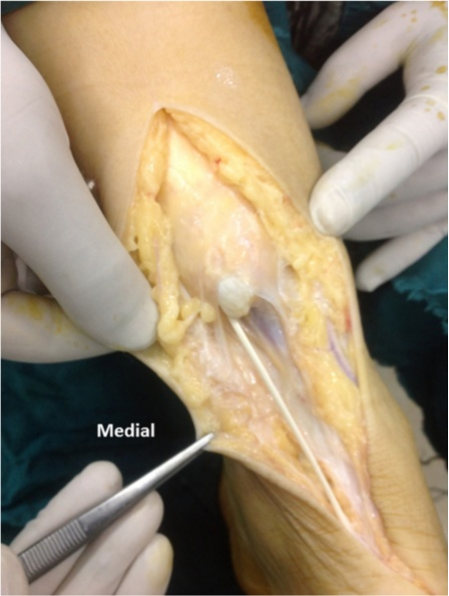
Intraoperative photo showing a large defect in the Achilles tendon (9 cm) with a rounded proximal stump and intact plantaris tendon

**Fig. 3:**
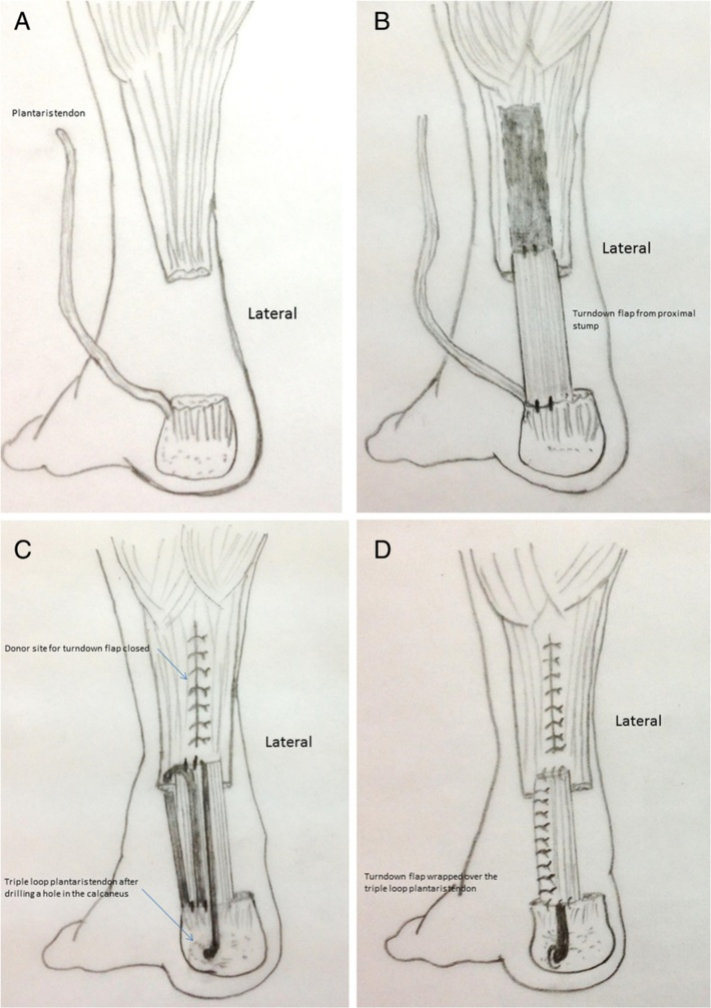
Schematic illustration of the surgical technique. **a** Diagram showing the magnitude of the Achilles tendon defect with the plantaris tendon being stripped from its origin keeping distal attachment intact. **b** Diagram showing the design of the turndown flap with its width being ½ the width of the proximal Achilles tendon stump and its length being 5 cm larger than the estimated defect. **c** Diagram showing the configuration of the triple-loop plantaris tendon after performing a drill hole in the calcaneus with the whole configuration lying in front of the turndown flap. **d** Diagram showing the final construct after wrapping the lateral half of the turndown flap over the plantaris triple loop

**Fig. 4:**
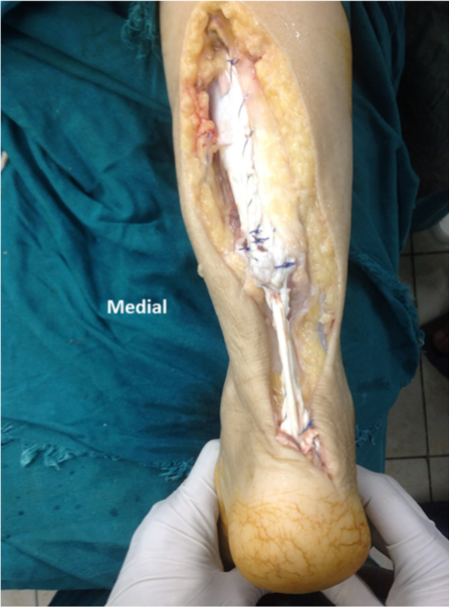
Intraoperative photo showing the final appearance of the Achilles tendon after reconstruction

Under spinal anaesthesia, patient was positioned prone with a pneumatic tourniquet applied at the midthigh. A midline 10–20 cm lazy S incision was performed starting just medial or lateral to the insertion of the Achilles tendon aiming proximally depending on the preoperative estimated defect. Both ends were routinely explored and debrided until healthy fresh tendinous tissues were encountered (Fig. 2). This was routinely followed by osteotomizing the Haglund’s prominence at the top posterior margin of the calcaneus. The length of the defect was measured after adequate mobilization of both tendon ends while the ankle was plantarflexed to 30° [8]. The ipsilateral plantaris tendon was routinely freed proximally using an open tendon stripper (Fig. 3a). A central turndown flap from the proximal portion of the Achilles tendon was raised distally based. The base of the flap was kept attached 2.5 cm from the proximal cut end to preserve sufficient reverse blood supply to the flap. The length of the flap was routinely 5 cm longer than the estimated defect to compensate for the overlap of the tendon at the proximal end and provide sufficient length for suturing to the distal end. The width of the flap was equal to the middle 2/4 of the proximal portion width (Fig. 3b). The flap was then turned down after tacking its medial proximal half with the proximal portion with running No. 1 Ethibond suture. The distal margin of the flap was then sutured to the distal end of the Achilles tendon at its medial half. The plantaris tendon was then looped in the coronal plane both in the proximal portion and through a drill hole in the calcaneus [via a 3.2-mm drill bit performing a tunnel for the tendon aiming form posteroinferior to anterosuperior] to form three loops connecting both ends (Fig. 3c). This was followed by wrapping the plantaris tendon loops with the lateral half of the turndown flap so that the smooth surface of the turndown flap faces the wound. Then the turndown flap was sutured to itself from proximal to distal with running No. 1 Ethibond suture (Figs. 3d and 4).

The proper tension of surgical reconstruction was assessed by both the degree of allowable passive dorsiflexion with the knee flexed to 90° and the presence of spontaneous active plantar flexion after forceful dorsiflexion of the ankle joint up to neutral position [8].

#### Postoperative care

**Fig. 5:**
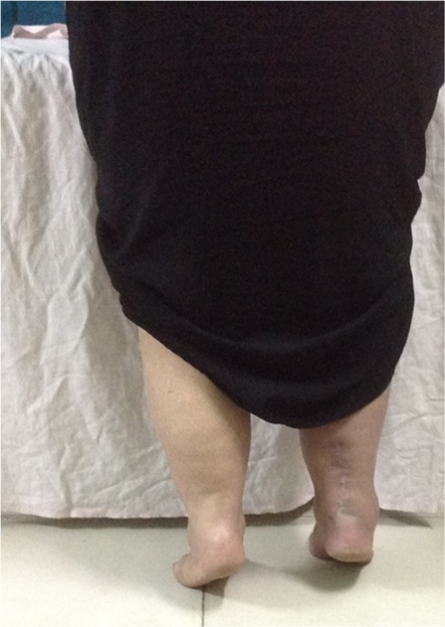
Postoperative photo (2 years) of a patient who underwent the proposed surgical technique with complete free tiptoe standing

The stitches were routinely removed on the 14th postoperative day. The operated leg was placed in a below-knee plaster cast with the ankle in gravity equinus for 4 weeks. This was followed by another below-knee plaster cast in the plantigrade position for 2 weeks. Finally, a short-leg walking cast was applied for 4 weeks. This was followed by nonsupported full-weight bearing (Fig. 5).

#### Statistical analysis

Data processing was performed using SPSS for Windows software (version 20). Average values were presented as means. Independent sample *t* test, Mann Whitney test, paired sample *t* test and Pearson’s correlation coefficient were used to evaluate the clinical and functional results. The results were considered statistically significant if a *P* value was <0.05.

#### Comparison within the MRI sessions after repair

To analyse the time course of the gap following surgery, the data from the first MRI session were compared with those from the second and third sessions using the Wilcoxon’s signed rank test. In addition, the paired data of the tendon gap disappearance rate between T1-weighted and T2-weighted images were also compared using the McNemar test.

## Results

The average period of follow-up was 21.8 months (range, 12–60 months). The patients’ preoperative and demographic data are depicted in (Table 1). The intraoperative data of all patients are shown in (Table 2).

**Table 1:** Patients’ demographic and anthropometric data

Patient (No.)	Age	Sex	Side	Time to interference (months)	BMI	Follow-up (months)
1	45	M	RT	6	33.06	60
2	48	F	RT	8	25.39	14
3	48	F	LT	8	25.39	14
4	29	F	RT	5	34.29	23
5	21	M	RT	1.5	24.84	12
6	32	F	RT	2	27.55	34
7	64	F	RT	3	35.92	19
8	35	M	LT	6	19	16
9	40	F	RT	4.5	25.95	26
10	50	F	LT	2	34.6	14
11	52	F	RT	5	37.03	24
12	48	F	RT	7	37.1	30
13	28	M	LT	10	31.14	23
14	45	F	RT	4	27.55	12
15	22	F	RT	12	21.48	13
16	38	F	RT	4	33.95	16
17	36	F	RT	2.5	36	20
18	52	F	RT	3	29.32	22
Mean	40.7			5.2	30	21.8

**Table 2:** Intraoperative data and complications

Patient (No.)	Level of rupture (cm)	Length of defect (cm)	Plantaris length (cm)	Turndown flap		Surgery time (minutes)	Complications
				Length	Width		
1	1.5	9	28	14	4	60	Superficial wound infection
2	1	7	27	12	3	45	
3	2	7	25	12	3	45	Delayed wound healing
4	2	8	26	13	3	50	
5	0.5	10	28	15	4	45	
6	3	7	24	12	3.5	50	
7	1.5	7	23	12	3.5	45	
8	2	8	29	13	3	45	
9	3	8	28	13	4	55	
10	3.5	8	26	13	3	50	
11	0.5	9	29	14	3	55	
12	2.5	7	25	12	3	50	
13	2.5	6	28	11	4	50	Delayed wound healing
14	3	7	25	12	3	55	
15	3.5	6	25	11	3	65	
16	4	10	28	15	3	60	
17	2	8	26	13	3.5	60	
18	2	7	26	12	3	50	
Mean	2.2	7.7	26.4	12.7	3.3	51.9	

The mean girth of the affected calf (39.2 cm) was less than that of the healthy calf (42.4 cm) with the mean difference being 3.3 cm (range, 1–6 cm).

The preoperative degree of active dorsiflexion of the affected side (mean = 21.2°) showed a statistically significant difference from both postoperative (mean = 19.4°) and healthy side (mean = 22.3°) values (*P* < 0.001, 0.002, respectively). In addition, the postoperative value showed a statistically significant reduction in comparison to the healthy side (*P* < 0.001).

The preoperative value of active plantar flexion (mean = 25.2°) showed a significant difference from both the healthy side (mean = 45.8°) and postoperative (mean = 43.1°) values (*P* < 0.001 in both). The postoperative values were much improved but showed a significant reduction in comparison to the healthy side (*P* < 0.001) (Table 3).

**Table 3:** Pre- and postoperative range of motion measurements and calf girth

Patients (No.)	Active plantar flexion			Active dorsiflexion			Affected calf girth	Healthy side girth
	Preoperative	Postoperative	Healthy side	Preoperative	Postoperative	Healthy side		
1	15	40	42	25	20	25	40	45
2	30	42	45	23	20	25	35	40
3	28	40	43	22	20	24	34	40
4	30	45	50	22	19	23	42	44
5	25	44	48	18	18	20	40	42
6	24	43	47	22	20	22	37	38
7	21	48	50	20	18	21	45	47
8	26	40	42	18	16	18	32	37
9	23	42	42	23	20	23	35	36
10	26	47	49	20	18	22	40	42
11	28	49	51	22	20	24	44	47
12	25	43	47	23	22	25	42	48
13	35	45	47	19	20	24	43	48
14	22	42	46	18	18	20	37	39
15	30	45	45	25	22	25	38	43
16	20	40	44	22	20	22	41	43
17	22	40	43	21	20	21	42	45
18	23	40	43	18	18	18	38	40
Mean	25.2	43	45.8	21.2	19.4	22.3	39.2	42.4
*P* value	Preoperative value in relation to sound side < 0.001*			Preoperative value in relation to sound side = 0.002*				
	Preoperative value in relation to postoperative value < 0.001*			Preoperative value in relation to postoperative value < 0.001*				
	Postoperative value in relation to sound side < 0.001*			Postoperative value in relation to sound side < 0.001*				

Postoperative measurements were recorded at 12 months postoperatively

*Indicates statistically signifcant values when *P* is < 0.05

The preoperative AOFAS ankle and hind foot score averaged 62.2 points while the postoperative scores averaged 86.8, 94.6 and 94.9 points at the 3rd, 6th and 12th months, respectively. The score exhibited both statistically significant improvement at both the 3rd and 6th months (*P* < 0.001 in both), and from the 3rd to the 6th month (*P* < 0.001). However, there was statistically non-significant improvement from the 6th to the 12th months (*P* = 0.331) (Table 4).

**Table 4:** Pre and postoperative AOFAS scoring

Patient (No.)	American Orthopaedic Foot and Ankle Society (AOFAS) ankle and hind foot scoring system			
	Preoperative	3 months postoperatively	6 months postoperatively	12 months postoperatively
1	42	82	89	89
2	47	90	100	100
3	47	82	92	98
4	85	99	100	100
5	76	89	100	100
6	75	88	95	95
7	51	82	89	89
8	41	84	95	95
9	70	85	92	92
10	58	85	93	93
11	73	85	90	90
12	72	87	97	97
13	40	83	93	93
14	70	85	95	95
15	51	93	100	100
16	65	85	95	95
17	85	95	95	95
18	72	83	93	93
Mean	62.2	86.8	94.6	94.9
*P* value	Preoperative value relation to 3rd and 6th months results (*P* < 0.001).			
	3rd to both the 6th and 12th months improvement (*P* < 0.001).			
	6th to the 12th months improvement (*P* = 0.331).			

There was no statistical significant relation between gender, body mass index (BMI), level of rupture or length of the defect and the postoperative scoring or ROM. However, using the Pearson’s correlation coefficient, there was a negative correlation between the length of the defect and both postoperative scoring and ROM. There was a statistically significant correlation between the lag of interference and both the value of postoperative active dorsiflexion and the calf atrophy (*P* = 0.025 and <0.001, respectively). There was a statistically significant correlation between age and the postoperative scoring at the 3rd, 6th and 12th months (*P* = 0.02, 0.004, and 0.013, respectively).

**Fig. 6:**
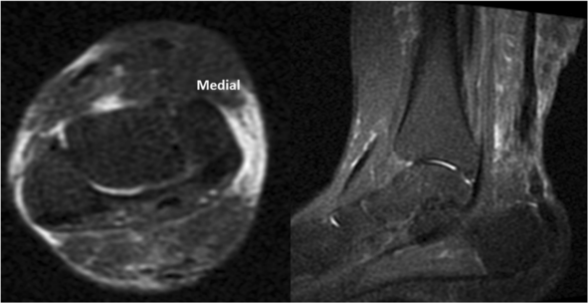
MR images obtained 28 days after surgery

**Fig. 7:**
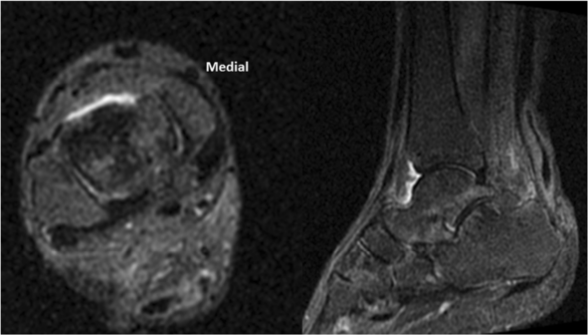
MR images obtained 58 days after surgery. Disappearance of tendon gap appears in T2 with fat sat.

**Fig. 8:**
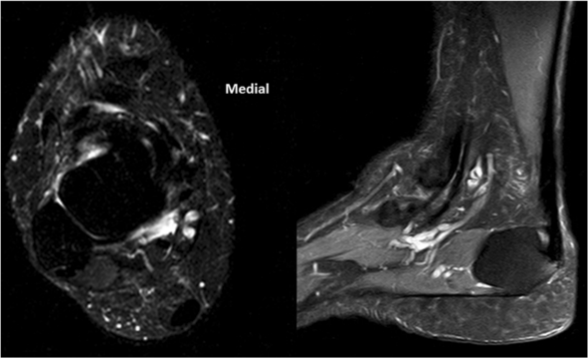
MR images obtained 85 days after surgery. Disappearance of tendon gap appears in T2 with fat sat.

At the first MRI T1-weighted images session, persistent Achilles tendon gap was depicted in all patients. T2-weighted images showed a tendon gap in 12 patients (66.7 %) (Fig. 6). The range of the gap length was 0.5–2.8 cm on T1-weighed images and 0–1 cm on T2-weighted images. While in the second MRI session, a tendon gap was found on T1-weighted images in two patients (11.1 %) while T2-weighted images showed no tendon gaps (Fig. 7). During the third MRI session, neither T1-weighted nor T2-weighted images showed a tendon gap in any case (Fig. 8). The percentages of cases of Achilles tendon compartment at the first, second, and third MRI sessions were 65, 90 and 100 % on T1-weighted images and 90, 100, and 100 % on T2-weighted images.

Disappearance of the tendon gap was observed significantly earlier on T2-weighted images (*P* = 0.063; significance level of *P* < 0.01). The length of the tendon gap during the second and third MRI sessions was significantly less than that during the first session (*P* < 0.005).

Three patients (16.7 %) developed minor complications in the form of superficial wound infection (one case) and delayed wound healing (two cases). All three cases were managed conservatively, employing daily dressing and antibiotics, within a 2-month period.

## Discussion

Reconstruction of a chronic Achilles tendon rupture poses a great challenge for orthopaedic surgeons which could be owed to the following: absence of consensus to the proper technique used, the anticipated large defect, muscle wasting, retraction, and short distal stump [3, 9].

Several techniques have been described in the literature for the management of such condition [5]. The primary goal of such techniques was restoration of both function and strength of the gastrocnemius-soleus complex by maintaining the optimal length-tension relationship [10]. In Myerson type III, the basic requirement is to bridge the defect by either biological tissue or synthetic material providing satisfactory strength that allows full range of tendon excursion. A number of options have evolved for such situation including either biological, synthetic, allogeneic tissues or combinations of any two of them [5, 6, 11, 12].

 Plantaris muscle tendon unit has been anatomically described either as a vestige or an accessory plantar flexor. The absence of this muscle is a common occurrence with a reported incidence of 8.2 % in males and 5.8 % in females [13]. The mean length of the plantaris tendon has been estimated at a recent anatomical study to be around 33 cm (range, 24–40 cm). In addition, there are many anatomical variations regarding the insertion of the plantaris tendon in the form of the following: (1) along with the Achilles tendon into the medial superior aspect of the calcaneus (50 %), (2) in front of the Achilles tendon into the top of the calcaneus (35.7 %), (3) fused with the Achilles tendon (9.5 %), or (4) into the deep fascia of the leg at the level of the ankle (4.8 %) [14].

The use of plantaris tendon as a tool for reconstruction of Achilles tendon has the advantage of being autogenous, easily harvested, together with the harmonious functional coherence. Its only drawback is the possibility of its absence in some patients [13, 14]. On the other hand, the use of turndown flap has been utilized by many authors and exhibited satisfactory results [5, 15].

Our new technique combined the advantages of using both the plantaris tendon and the turndown flap from the proximal portion of the Achilles tendon. This technique has the following advantages: (1) single incision, (2) synergistic function of the construct, (3) increased cross-sectional area of the tendon as a result of using triple-loop plantaris and double-thickness turndown flap, (4) preservation of some vascularity in the utilized grafts, and finally, (5) provision of smooth surface of the turndown flap that preserves smooth gliding of the construct which subsequently minimizes postoperative adhesions. The authors believe that this technique is very suitable for patients having chronic ruptures of the Achilles tendon Myerson type III who have plantaris tendon and a good skin condition. In addition, when the allografts or synthetic tapes are not available, this technique will be a good reconstructive option.

On the other hand, this technique is contraindicated in patients who have no plantaris tendon or have bad skin or peripheral vascular status. The disadvantages of this technique are mainly as follows: (1) the necessity of existence of plantaris tendon, (2) the difficulty of the technique, and (3) the possibility of skin breakdown or infection.

Complications of the Achilles tendon reconstruction include wound dehiscence, weakness, decreased ankle ROM, calf atrophy, sural nerve injury, and re-rupture [16]. In our series, we were confronted with (16.7 %) incidence of complications in the form of wound-healing problems or superficial wound infection with no other major complication.

Jain et al. [5] had proposed a similar technique for the management of chronic Achilles tendon ruptures with defects longer than 2 cm. However, they utilized free plantaris tendon graft with a circular configuration enveloping the turndown flap.

Magnetic resonance imaging can delineate the condition of the ends of completely ruptured tendons. A normal Achilles tendon is viewed as an area of low signal intensity on all sequences. Chronic ruptures are seen as an area of low signal intensity on T1-weighted images and as discontinuity and an altered signal on T2-weighted scans [17]. An area of hyperintensity at the rupture site on T2-weighted images was visualized 3 weeks after open surgical repair by some authors which they considered active scar tissue [18, 19].

The tendon gap had disappeared on MRI 11–13 weeks after surgery which was not correlated with the gap length determined on MR images. This observation suggests that tendon continuity may be restored rapidly in the advanced stage of healing because repair takes place over the entire tendon gap simultaneously. Early postoperative palpation did not deny the presence of a tendon defect that may be visible as a tendon gap during the second MR session, which could be explained by the fact that this gap on MR images might already have been filled with granulation tissue whereas mature fibrous tissue may still be absent [16] in addition to the rigid platform provided by our technique.

We found that postoperative MRI of the Achilles tendon revealed a tendency for earlier disappearance of the tendon gap on T2-weighted than on T1-weighted images. This reflects the higher sensitivity of T2-weighted imaging in perceiving the transition of blood-rich immature granulation tissue to fibrous scar tissue. In our study, restoration of the compartment of the Achilles tendon preceded disappearance of the tendon gap.

We believe that MRI is the best noninvasive method for evaluating postoperative changes in the Achilles tendon, but a minor limitation was present because the measurement used in this study would likely provide greater potential measurement error than ±2 mm. Other limitations of this study are the lack of histologic correlation of the tendon gap seen on MR images which can clarify healing of the ruptured Achilles tendon and the small number of patients.

## Conclusions

This study presents a new technique for reconstruction of chronic ruptures of the Achilles tendon Myerson type III. We believe that this technique is biologic, synchronous and reliable. The results of this study confirmed that the Achilles tendon healing together with tendon gap disappearance have been perceived with higher sensitivity in T2-weighted images than in T1-weighted images.

## Abbreviations

AOFAS: American Orthopaedic Foot and Ankle Society
BMI: body mass index
ROM: range of motion
